# Salvianolic Acid B Inhibits Hand-Foot-Mouth Disease Enterovirus 71 Replication through Enhancement of AKT Signaling Pathway

**DOI:** 10.4014/jmb.1907.07079

**Published:** 2019-11-11

**Authors:** So-Hee Kim, Jihye Lee, Ye Lin Jung, Areum Hong, Sang-Jip Nam, Byung-Kwan Lim

**Affiliations:** 1 Department of Biomedical Science, Jungwon University, Goesan-gun, Chungbuk 28024, Republic of Korea; 2 Department of Chemistry and Nanoscience, Ewha Womans University, Seoul 10-750, Republic of Korea; 3 Graduate School of Industrial Pharmaceutical Sciences, Ewha Womans University, Seoul 120-750, Republic of Korea

**Keywords:** Salvianolic acid B, enterovirus 71, myocarditis, apoptosis, hand-foot-mouth disease

## Abstract

Hand, foot, and mouth disease (HFMD) is caused by enterovirus 71 (EV71) in infants and children under six years of age. HFMD is characterized by fever, mouth ulcers, and vesicular rashes on the palms and feet. EV71 also causes severe neurological manifestations, such as brainstem encephalitis and aseptic meningitis. Recently, frequent outbreaks of EV71 have occurred in the Asia-Pacific region, but currently, no effective antiviral drugs have been developed to treat the disease. In this study, we investigated the antiviral effect of salvianolic acid B (SalB) on EV71. SalB is a major component of the *Salvia miltiorrhiza* root and has been shown to be an effective treatment for subarachnoid hemorrhages and myocardial infarctions. HeLa cells were cultured in 12-well plates and treated with SalB (100 or 10 μg/ml) and 106 PFU/ml of EV71. SalB treatment (100 μg/ml) significantly decreased the cleavage of the eukaryotic eIF4G1 protein and reduced the expression of the EV71 capsid protein VP1. In addition, SalB treatment showed a dramatic decrease in viral infection, measured by immunofluorescence staining. The Akt signaling pathway, a key component of cell survival and proliferation, was significantly increased in EV71-infected HeLa cells treated with 100 μg/ml SalB. RT-PCR results showed that the mRNA for anti-apoptotic protein Bcl-2 and the cell cycle regulator Cyclin-D1 were significantly increased by SalB treatment. These results indicate that SalB activates Akt/PKB signaling and inhibits apoptosis in infected HeLa cells. Taken together, these results suggest that SalB could be used to develop a new therapeutic drug for EV71-induced HFMD.

## Introduction

Salvianolic acid B (SalB) is a major component of the *Salvia miltiorrhiza* root, which belongs to the plant family Labiatae. This compound is widely used in traditional Chinese medicine for the treatment of various cardiovascular diseases and has been reported to have potential protective effects from oxidative injury [1].  Furthermore, it has been shown to have protective effects on the liver [2, 3], kidneys, and lungs, particularly improving ischemia-/reperfusion-(I-/R-) induced injury [4, 5]. In traditional Chinese medicine, SalB is used for improving blood circulation to clear blood stasis, regulating menstruation, and as an analgesic to treat heartburn and anxiety [6]. However, the antiviral effects of SalB are not well understood. According to the pharma-cological structure of phenolic acid compounds, salvianolic acid belongs to the group of polyphenolic acids, which includes compounds such as rosmarinic acid, lithospermic acid, and other salvianolic acids.

Human Enterovirus 71 (EV71) belongs to the enterovirus group of the Picornaviridae family and is one of the major causative agents of hand, foot, and mouth disease (HFMD)[7]. EV71 infection is associated with severe neurological diseases such as acute encephalitis and flaccid paralysis in infants and children under six [8, 9]. EV71 is a positive-sense, single-stranded RNA virus and consists of a single open reading frame flanked by 5’ and 3’ untranslated regions (UTRs). The 5’-UTR includes an internal ribosome entry site (IRES), which initiates the synthesis of the viral polyproteins (VP1-VP4) and non-structural proteins (2A-2C and 3A-3D) [10]. The majority of antiviral drugs target a specific component of the virus. The high replication and mutation rates of enteroviruses can result in the virus developing resistance to this type of antiviral treatment. Targeting host factors such as cell signaling molecules and apoptosis inducers may establish a higher genetic barrier to resistance and can be used in combination with viral inhibitors to treat these infections [11]. To identify novel compounds to combat enterovirus infections, we tested the ability of naturally occurring products to modulate cell signaling and inhibit viral replication. In this study, we have discovered that SalB has potent antiviral effects on enterovirus EV71. SalB inhibited proliferation of EV71 in HeLa cells and activated the Akt signaling pathway, which promotes cell survival and proliferation. In addition, SalB reduced apoptosis and production of the viral capsid protein VP1. These results demonstrated that SalB may be a useful compound in the development of an antiviral treatment for EV71 infections.

## Materials and Methods

### Virus and Cell Lines

EV71 was cultured on HeLa cell monolayers. HeLa cells grown for 16 h were infected with 10^7^ plaque-forming units (PFU) of EV71. When the cytopathic effect (CPE) of the infected cells reached > 90%, the cells were subjected to three freeze-thaw cycles at -80°C. Virus stock concentrations were determined by tissue culture infectious dose 50 (TCID50). HeLa cells were cultured using Dulbecco’s Modified Eagle Medium (DMEM) with 5% fetal bovine serum at 37°C in a humidified 5% CO_2_ incubator [12].

### Purification of Salvianolic Acid B

Dried danshen roots (5.4 kg) were extracted three times with methanol (MeOH) at room temperature and the solvent was removed with a vacuum. The extract was partitioned between dichloromethane and water (H_2_O). The aqueous layer was adjusted to pH 2.0 with addition of trifluoroacetic acid and was further partitioned with ethyl acetate (EtOAc). The EtOAc-soluble layer was fractionated by flash silica column chromatography and eluted with a step gradient of chloroform, MeOH and H_2_O (3:1:0.1→1:1:0.1, v/v/v). The fraction containing the mixture of salvianolic acids was further isolated by reversed-phase HPLC to isolate salvianolic acid B (100 mg). Based on a comparison of the MS and NMR data with previously reported data, compound 1 was identified as salvianolic acid B [16]. The purity of SalB was 96.7 ± 0.2% using area normalization methods by HPLC-UV.

### Defining Antiviral Effect 

To identify a compound with significant antiviral activity, HeLa cells cultured on a 96-well plate were infected with 20 µl of EV71 (10^6^ PFU/ml) and treated with compound, which were serially diluted from 100 µg/ml to 1 ng/ml. At 16 h post-infection, cell survival rate was measured with addition of 8 µl of Cell Counting Kit 8 (CCK-8; Dojindo Molecular Technologies, Inc., USA) reagent [13].

### Virus Infection and Salvianolic Acid B Treatment

To observe whether SalB modulates Akt/PKB signaling, infected HeLa cells were treated with purified SalB on a 12-well plate. First, HeLa cells were infected at a multiplicity of infection (MOI) of 1.0 in all wells except the negative control. SalB was serially diluted from 100 µg/ml to 1 ng/ml in Dulbecco’s Modified Eagle Medium (DMEM) with 5% Fetal Bovine Serum (FBS) and treated to the cells after 0.5 h infection. At 16 h post-infection, cell survival and cytotoxicity were measured by CCK-8 [13, 14] or protein was extracted for western blot analysis.

### Western Blot Analysis

Total protein from SalB-treated HeLa cells was extracted and analyzed by western blot. Protein was extracted using 1X PBS lysis buffer (1X PBS, 0.5 mM EDTA, 0.1% Triton X-100, and Protease inhibitor cocktail) mixed with sample buffer. Aliquots of total cell extracts were loaded onto 10% acrylamide gel. After electrophoresis, the cells were transferred to Hybond-ECL PVDF membrane. The membranes were blocked with 5% non-fat milk blocking buffer and probed with eIF4G1 (1:1000, rabbit polyclonal antibody), p-Akt (1:1000, mouse monoclonal antibody), total-Akt, and enteroVP-1 antibody (1:1000, mouse monoclonal antibody)[14]. All data were quantified by NIH-ImageJ software [15].

### Immunofluorescent Stain

EV71 proliferation was confirmed by immunofluorescent stain of capsid protein VP1. HeLa cells were infected by EV71 with diluted SalB. After 16 h, HeLa cells were fixed by cold methanol for 15 min, followed by blocking and permeabilization with 2%Bovine serum albumin (BSA) and 0.2% Triton X-100 in PBS and incubated with primary antibodies as follows: rabbit enterovirus 71 VP-1 antibody (Merck Millipore, Germany). Target proteins were visualized with secondary antibodies conjugated with fluorophores (Alexafluor 488, 1:250; Merck Millipore) and Hoechst nuclear stain. Fluorescence images were taken and processed using a fluorescent microscope (Olympus Co., Japan).

### Reverse Transcription PCR 

To analyze gene expression, total RNA was isolated from EV71-infected and non-infected HeLa cell by TRIzol reagent (Thermo Fisher Scientific, USA). The reverse-transcription reaction was performed using the Maxime reverse-transcription (RT) kit (Intron Biotech, Inc., Korea) with 2 µg of total RNA as the template. Apoptosis and cell cycle regulation were measured by amplification of Bcl-2 and Cyclin D1, respectively. PCR with the synthesized cDNA was performed with primers for Bcl-2 sense primer (5’- AATGAACTCTTTCGGGATGG-3’), Bcl-2 antisense primer (5’-CCAACTTGCAATCCGACTCA-3’), CyclinD1 sense primer (5’-AACTACCTGGACCGCTTCCT-3’), CyclinD1 antisense primer (5’-CCACTTGAGCTTGTTCACCA-3’), GAPDH sense primer (5’-ATCAACGACCCCTTCATTGAC-3’), and GAPDH antisense primer (5’-CCAGTAGACTCCACGACATACTCAGC-3’) with cDNA as template. Then, the PCR product was electro-phoresed on 1.5% agarose gel. All data were quantified by NIH-imageJ software [14].

### Statistical Analysis

All data were expressed as mean ± SEM. The results of Control and Virus Infection groups were analyzed by Student *t*-test by Prism 3.0. Statistically, *p* < 0.05 was considered significant.

## Results

### Antiviral Effect of Salvianolic Acid B 

**Fig. 1:**
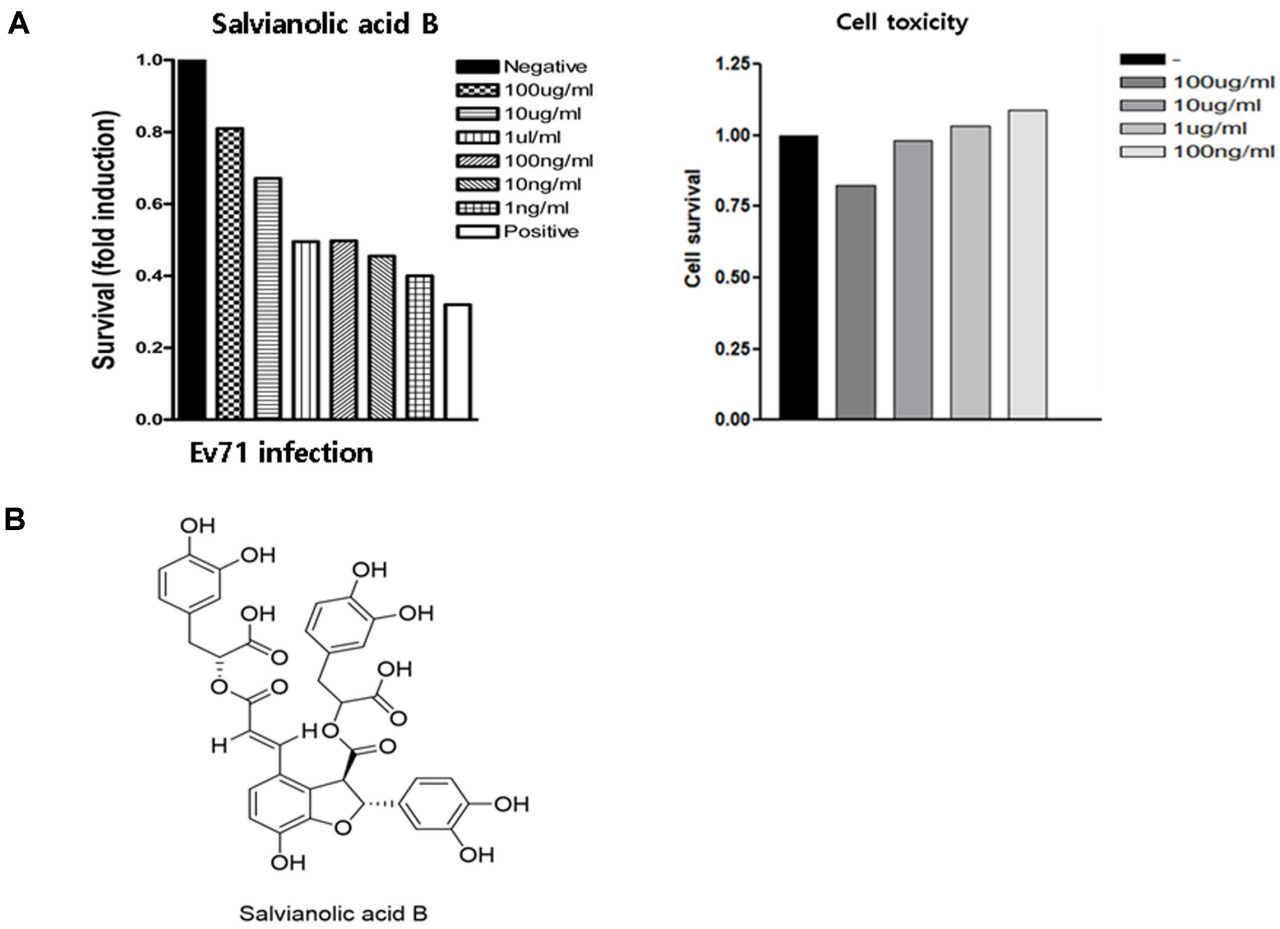
Salvianolic acid B has potent antiviral activity. (**A**) The antiviral effect of SalB was assessed by cell survival of EV71-infected HeLa cells (left panel). negative: without virus infection, positive: virus infection only. Cytotoxicity was measured with a CCK-8 cell survival assay (right panel). Data shown by the fold induction. (**B**) Chemical structure of Salvianolic acid B.

The antiviral effect of SalB against EV71 was analyzed by measuring the survival of infected HeLa cells with and without SalB treatment. Infected HeLa cells were treated with a range of SalB concentrations from 100 µg/ml to 1 ng/ml. The survival rate of HeLa cells increased with treatment of 10 µg/ml or greater of SalB compared to the untreated sample. Moreover, the cytotoxicity of HeLa cells was reduced with SalB treatment in a dose-dependent manner. Cell death was not observed in cells treated with a high concentration of SalB (Fig. 1A). The chemical structure of SalB is shown in Fig. 1B.

### SalB Inhibits EV71 Protein Production 

**Fig. 2:**
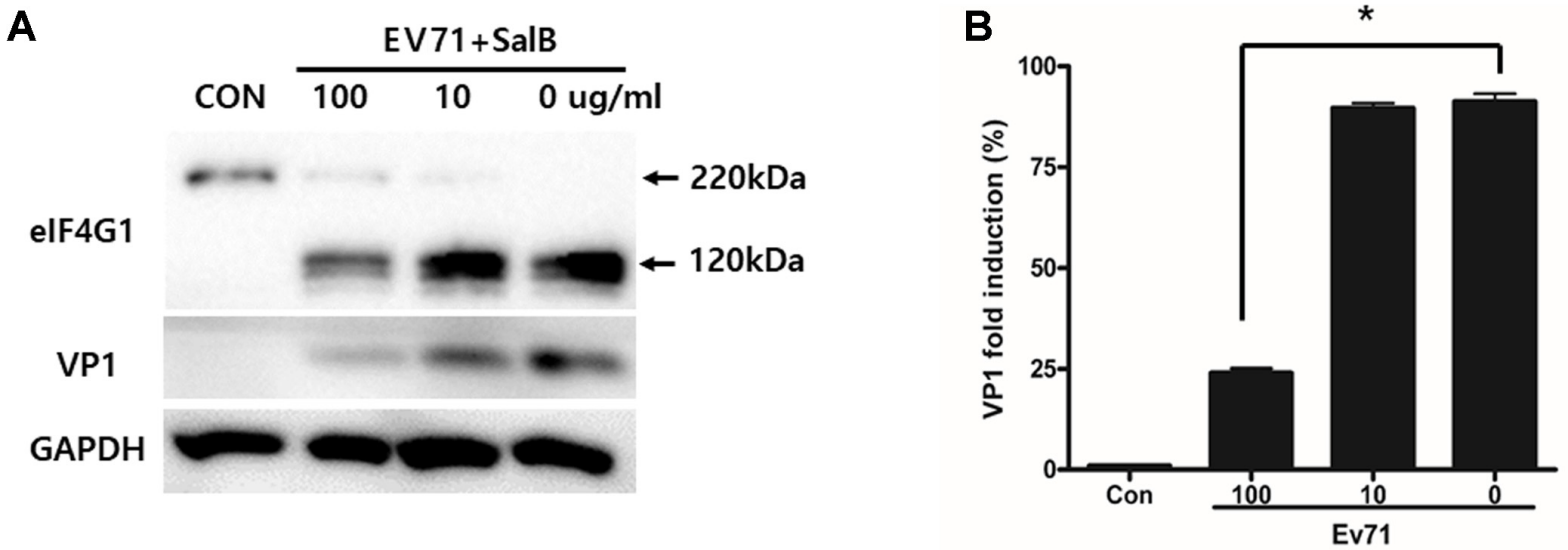
SalB inhibits EV71 replication and protease 2A production. (**A**) Addition of 100 μg/ml SalB to EV71-infected HeLa cells significantly decreased eIF4G1 cleavage by the viral protease 2A. (**B**) SalB treatment of infected HeLa cells reduces the production of the VP1 protein. Western blot results were quantitated by NIH-ImageJ. All data are presented by the mean ± SEM, *p < 0.05 by two-tailed Student’s *t*-test.

The replication of EV71 was assessed by measuring the expression of the viral capsid protein VP1 by western blot analysis. VP1 expression was significantly reduced by 75%after treatment with 100 µg/ml SalB (Fig. 2). At this concentration, activity of the viral protease 2A was also reduced, as measured by western blot analysis of the eukaryotic transcription initiation factor eIF4G1 cleavage (Fig. 2A). The western blot data was quantified by ImageJ software and represented by graph (Fig. 2B).

### SalB Attenuates EV71 Replication

**Fig. 3:**
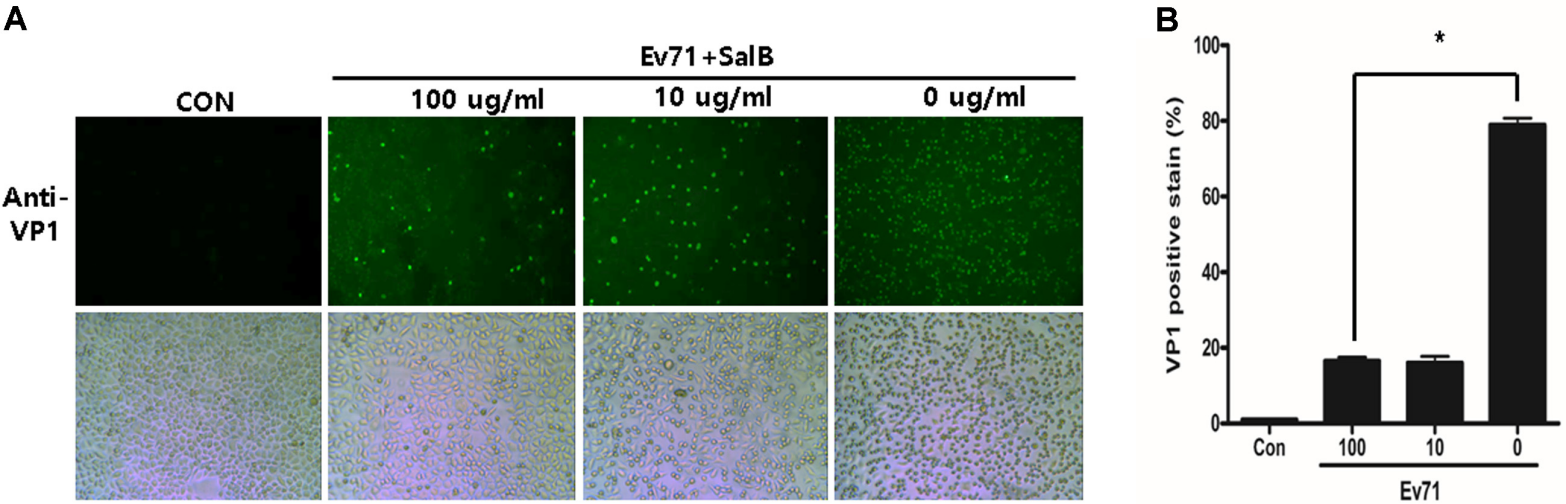
SalB inhibits EV71 capsid protein production. (**A**) SalB treatment of EV71-infected HeLa cells reduced the expression of VP1. The bright field pictures showed the cytopathic effect by EV71 infection. (**B**) Immunofluorescent stain results were quantified by NIH-ImageJ. All data are presented by the mean ± SEM. *p < 0.05 by two-tailed Student’s *t*-test.

We measured EV71 replication by immunofluorescent staining of the viral capsid protein VP1. EV71-infected HeLa cells were treated with 100 and 10 µg/ml SalB. Cells were then stained with an anti-VP1 antibody and capsid proteins were visualized using a fluorescence microscope (Fig. 3A). The amount of HeLa cells expressing VP1 was significantly reduced in SalB-treated cells in a dose-dependent manner (Fig. 3).

### SalB Inhibits Apoptosis through Akt Activation in EV71 Infection

**Fig. 4:**
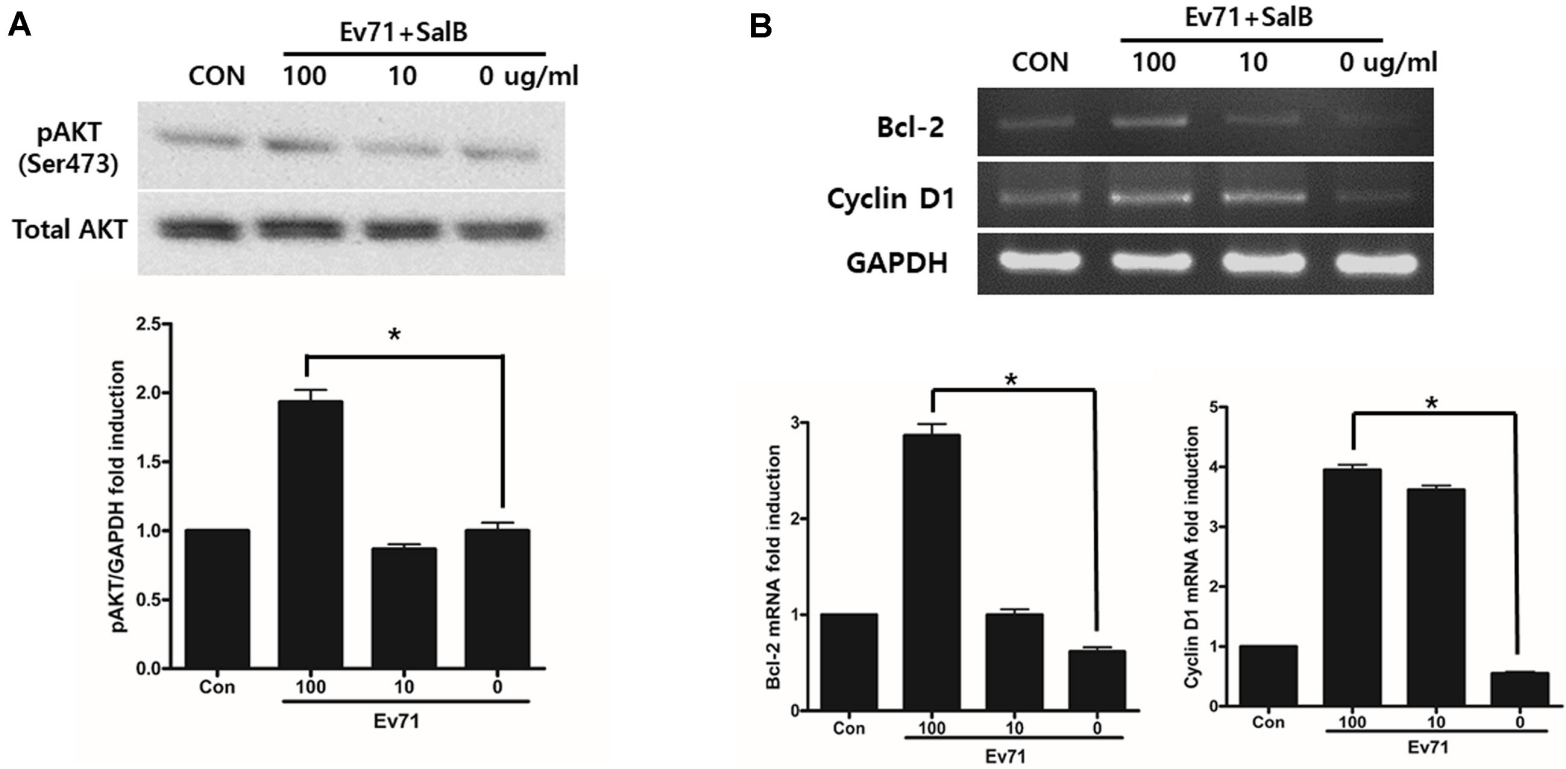
SalB inhibits early replication of EV71 and infected cell apoptosis. (**A**) Total protein from EV71-infected cells treated with SalB was subjected to western blot analysis using the phospho-Akt (Ser473) antibody. (**B**) The anti-apoptotic gene Bcl-2 and cell cycle regulator Cyclin-D1 were significantly increased in cells treated with 100 μg/ml SalB. All data are presented by the mean ± SEM of 3 independent experiments. *p < 0.05 by two-tailed Student’s *t*-test.

In previous studies, enteroviruses have been reported to increase the activity of the Akt signaling pathway at late time points [11]. These findings suggest that this pathway may be important for EV71 proliferation after the initial infection. Therefore, SalB’s inhibitory effect on EV71 replication may be through modulation of the Akt signaling pathway. Alterations in cell signaling in EV71-infected cells treated with SalB were analyzed by western blot. Akt phosphorylation increased significantly when cells were treated with 100 µg/ml of SalB compared to the control (Fig. 4A). To observe whether inhibition of apoptosis is due to Akt activation, we measured Bcl-2 and cyclin-D1 mRNA levels by RT-PCR. Expression of both genes increased significantly when 100 µg/ml of SalB was added to EV71-infected cells. These results suggested that treatment of SalB significantly inhibited the apoptosis signal by through Akt activation at an early time point of EV71 infection.

## Discussion

Enterovirus 71 (EV71) has a genome structure and life history with similar replication cycles to the characterized polioviruses, and the same method of vaccine development has been used for both groups. However, effective vaccines for EV71 have not been developed [10]. In this study, we have discovered and characterized the antiviral activity of SalB against EV71.

EV71 infections are associated with severe neurological diseases such as acute encephalitis and flaccid paralysis in infants and children under the age of six. Additionally, it is an aberrant inflammatory process that causes HFMD [8, 9]. We tested the inhibitory effect of SalB on EV71 infection. We found that treatment with SalB significantly suppressed EV71 infection and increased cell survival. We also observed that SalB reduced damage to HeLa cells by inhibiting virus capsid protein (VP1) production. The cleavage of eIF4G1, a transcription initiation factor that is cleaved by viral protease 2A [15], was significantly decreased by SalB treatment compared to the no treatment control samples.

Previous study showed that Akt is an important signaling molecule that regulates coxsackievirus B3 (CVB3) replication. At 8 h post-infection with CVB3, inhibition of Akt leads to effective attenuation of viral replication [11]. We discovered that Akt signaling is also an important target to inhibit EV71 replication. However, its role in this process is unique from what has been shown for CVB3. SalB treatment strongly activates Akt, leading to down-regulation of apoptosis through the induction of the anti-apoptotic Bcl-2 pathway. Induction of Akt signaling at early time points in infection caused a significant decrease in the production of viral proteins VP1 and activity of protease 2A. However, the specific mechanism of this process remains unclear. More studies will have to be done to further characterize the link between apoptosis and EV71 replication.

In conclusion, we found that SalB activates Akt signaling and the expression of the anti-apoptotic Bcl-2 pathway. It down-regulates apoptosis in EV71-infected HeLa cells and inhibits EV71 replication. These results, therefore, suggest that SalB may be a useful compound when developing new therapeutic agents to treat HFMD.
